# The Myriapoda of Halimun-Salak National Park (Java, Indonesia): overview and faunal composition

**DOI:** 10.3897/BDJ.7.e32218

**Published:** 2019-04-15

**Authors:** Michael Hilgert, Nesrine Akkari, Cahyo Rahmadi, Thomas Wesener

**Affiliations:** 1 Rheinische Friedrich-Wilhelms-Universität, Bonn, Germany Rheinische Friedrich-Wilhelms-Universität Bonn Germany; 2 Naturhistorisches Museum Wien, Wien, Austria Naturhistorisches Museum Wien Wien Austria; 3 Indonesian Institute of Sciences, Cibinong, Indonesia Indonesian Institute of Sciences Cibinong Indonesia; 4 Indonesia Speleological Society, Bogor, Indonesia Indonesia Speleological Society Bogor Indonesia; 5 Zoological Research Museum A. Koenig, Leibniz Institue for Animal Biodiversity, Bonn, Germany Zoological Research Museum A. Koenig, Leibniz Institue for Animal Biodiversity Bonn Germany

**Keywords:** Biodiversity, Chilopoda, Diplopoda, Indonesia, Java, Halimun-Salak National Park, South-East Asia

## Abstract

The myriapod fauna of the mega-diverse country of Indonesia is so far insufficiently known, with no species lists or determination keys. In order to obtain an overview of the faunal composition of the Myriapoda in an Indonesian forest system, the fauna of the Halimun-Salak National Park in western Java was explored during the dry season (September–October 2015) in the framework of the German-Indonesian INDOBIOSYS project (Indonesian Biodiversity Discovery and Information System). A total of 980 Myriapoda specimens were collected by hand by 3–4 researchers from three different sites in the national park, from which 796 specimens were determined to a higher taxonomic level (class, order, family) and 617 specimens were determined to morphospecies. Among these, 27 were Symphyla (4%) (excluded from further analyses), 226 Chilopoda (28%) and 543 Diplopoda (68%). The Scolopendromorpha (64% of all identified centipedes) and Polydesmida (69% of all identified Diplopoda) were the most represented orders in our samples.

Twenty-four morphospecies of Chilopoda were determined: one each of Scutigeromorpha and Lithobiomorpha, six Scolopendromorpha and sixteen Geophilomorpha. Nine orders of diplopods were present, with a total of 47 morphospecies: one each of Polyxenida, Glomeridesmida and Chordeumatida, two each of Glomerida, Spirobolida and Siphonophorida, seven of Sphaerotheriida, ten of Spirostreptida and 21 of Polydesmida.

Two species curves were obtained to have a first idea about the myriapod diversity in the Halimun-Salak National Park and to compare the three individual collecting sites.

Our results depict the Scolopendromorpha as the most common centipedes in Javanese rainforests and the Geophilomorpha as the most species-rich order. In contrast, the Polydesmida were the most dominant millipede group with 167 specimens and with 13 morphospecies the family Paradoxosomatidae was the most diverse.

## Introduction

With ca. 12,000 described species (Kime and Enghoff 2017), Diplopoda (millipedes) is the most species-rich class of the subphylum Myriapoda. The group encompasses macroinvertebrate soil organisms and plays an important role in nutrient recycling (Hopkin and Read 1992). Whereas the myriapods conquered nearly every terrestrial territory (Kime 2000), the millipedes show a strong affinity to the leaf-litter layer in broadleaf- and mixed-deciduous forests (Dunger 1993). Most millipedes are decomposers and eat dead wood, leaf litter, or other dead organic material like algae (Polyxenida) and fungi (Platydesmida) (Sierwald and Bond 2007).

Despite the continuous and increasing interest in the myriapod fauna of SE Asia (e.g Golovatch 2018, Silva et al. 2017, Zoysa et al. 2016, Siriwut et al. 2015, Vahtera and Edgecombe 2014), our knowledge on the actual diversity of millipedes in the tropical region remains fragmentary, with no more than 10% of its total diversity hitherto described. For instance, the Diplopoda fauna of India includes only 270 described species (Golovatch and Wesener 2016), showing a clear sign of the lack of studies as the better known Diplopoda fauna of Italy alone encompasses almost twice as many (470) known species (Strasser and Minelli 1984).

Lying on both sides of the equator, Indonesia is part of one of the most important biodiversity hotspots in the world (Myers et al. 2000, Norman 2003). This is also true for millipedes as 14 of the 16 known orders were hitherto recorded from the country, making it home to one of the most diverse millipede faunas at a higher systematic level (Golovatch and Shelley 2011). This fauna is unfortunately under a continuous threat of decline, and in some cases even extinction, due to a growing anthropogenic pressure in relation to agricultural activities and other socio-economic driven factors. Among these, destruction of natural habitats and conversion of natural forests to palm oil plantations is a significant threat to biodiversity (Prasetyo et al. 2009). This is especially prevalent on the island of Java, one of the most densely populated areas on this planet (Goldewijk 2005).

While complete Myriapoda inventories at the species level exist for the Cat Tien National Park in Vietnam (Golovatch et al. 2011) and for Singapore (Decker 2013), no other particular site in Asia has been inventoried completely and no single publication had looked at the diversity of different orders and families of myriapods in an Asian forest.

The aim of this research was to provide a first overlook and a quantitative species list of centipedes and millipedes for rainforests in tropical Asia found in a given amount of time during the dry season, namely in the Halimun-Salak National Park, and to determine which orders, families or (morpho-)species were the most diverse. For this, the fauna of the Halimun-Salak National Park in western Java was explored by a team of German biologists during the dry season (September–October 2015) in the framework of the German-Indonesian INDOBIOSYS project. The collected material was later identified to morphospecies and higher levels, and four species accumulation analyses were performed. The class Symphyla was only included in the class-level statistics as morphospecies determination was not attempted.

## Material and Methods

### Collecting sites and habitats

The material was hand-collected by Thomas Wesener and Jan Philip Oeyen in the Halimun-Salak National Park during the dry season, between the 9th of September and the 7th of October 2015 (29 days) with the help of local guides and an Indonesian research assistant.

Three different habitats and sites were explored:

1. Mt. Salak and Sukamantri in the eastern area around the volcano,
2. Cidahu below the mountain, and
3. Cikaniki in the centre of the National Park, where the Research Station was located.

See Fig. 1.

These areas are exposed to different degrees of human influence, which is reflected in their vegetation.

Habitat 1, Mt. Salak, had only little natural vegetation left, which was dominated by banana plantations and *Casuarina* forests.

Habitat 2, Cidahu, was semi-natural. It was a former deforested area and has now been regenerating for several years. On the one hand there were some freshly regrown indigenous rainforest parts, on the other hand some alien species and even some neophytes like bananas (*Musa*) and Australian *Casuarina* had started to spread within the floral compositions.

Habitat 3, Cikaniki, was the only near-natural area. It had primary rainforests and only selective logging was found at some places.

Every single collection event in the three habitats received its own identification number (labelled “HAL XXX” for Halimun-Salak National Park).

### Sampling techniques

All specimens were collected by hand and preserved in 96% ethanol. Quantitative sampling was not possible as sieves and Winkler extractors were retained in customs.

Every habitat received a different amount of collecting effort of 10 hours per day per person. Night catches added 4 more hours per worker to the workload. Habitat 3, Cikaniki received the highest amount of sampling hours and was the only collection site with night catch events.

The specimens were sorted and all the individual sample glasses got voucher labels (e.g. HAL45-02). Each label stated the voucher number, the HAL number, the exact location with the GPS data and the date when the sample was collected.

For a better and quicker identification, photos of millipedes were taken with a Leica Z6APO Q Imaging System (Q Imaging, Canada; www.qimaging.com) together with the programme Auto-Montage Pro 5.03.0061 from the company Synoptics (Cambridge, Great Britain 2006; http://www.synoptics.co.uk). The pictures were edited in Adobe Photoshop CS2 9.0.2.

Sometimes, large amount of juveniles were collected at a single collection event (probably nests of freshly hatched eggs). To avoid a strong bias caused by these random findings, only three specimens of those nests were included in the data analyses.

It was not possible to identify the juveniles to species-level (or even genus- or family-level). Thus, unidentified juveniles were not included in the data analyses at the species- or generic-level. The collected material will be shared between Museum Koenig, Bonn, Germany and LIPI, Bogor, Indonesia.

### Species identification

As no determination keys for Indonesian millipedes exist, numerous encountered species might be undescribed, and most of the described Indonesian Myriapoda genera and species are in need of revision. Therefore, we chose the morphospecies approach. In several cases, especially for numerous Polydesmida and Geophilomorpha, a trustworthy determination to genus level was not possible. In a few cases, a determination to family level was not possible for the Spirostreptida. Those specimens were labelled as “Spirostreptida Family 1 Genus A spec. 1”. For a complete list of determinations, refer to the supplementary material (Suppl. material 1).

For diverse groups outside our area of expertise, the identifications were verified by international experts. The scolopendromorphs were, for instance, identified by Arkady Schileyko (Moscow Lomonosov State University, Moscow), while the polydesmidans were identified by Sergei Golovatch (Russian Academy of Sciences, Moscow) and Weixin Liu (South China Agricultural University, Guangzhou).

The morphospecies determination for the genus *Otostigmus* (Scolopendromorpha, Chilopoda) was problematic. Only identification to species-groups (after Lewis 2010) was possible, likely leading to an underestimation of the actual number of *Otostigmus* species in our samples.

### Data analyses

After the identification of all specimens to class, order, family, (morpho-)genus and morphospecies, they were sorted in a chart in Microsoft Excel 2010 (Microsoft Corporation) for the data analyses. Symphyla were removed from all except the class-level statistics, as morphospecies determination was not attempted.

For the specimen-level analyses, juveniles (except when more than 3 were collected in a swarm, see above) were retained, whereas they were removed from the analyses at the morphospecies-level. 769 specimens (226 Chilopoda and 543 Diplopoda) were available for the specimen analyses, while 617 specimens could be incorporated in the morphospecies analyses.

A species accumulation analysis was performed with the software “environment for statistical computing and graphics R” version 3.3.0 (03.05.2016) developed by the R-Core Team in 1993 (https://www.r-project.org) (*Ihaka and Gentleman 1996*). Four species accumulation curves were obtained.

## Results

### Faunal composition of Halimun-Salak National Park

Of a total of 980 collected specimens, excluding a high number of juveniles, 796 Myriapoda samples from the Halimun-Salak National Park were analysed and 617 samples were identified to morphospecies (Figs 2, 3, 4). Nearly two-thirds (543 samples) were determined as Diplopoda. A little more than one-quarter (226 samples) of the remaining Myriapoda were identified as Chilopoda. A small fraction of 4% (27 samples) belonged to the class Symphyla, which was excluded from the study.

### Chilopoda - centipedes

The samples revealed seven different families of the orders Scutigeromorpha, Lithobiomorpha, Scolopendromorpha and Geophilomorpha.

The majority of the Chilopoda collected at Halimun-Salak (145 individuals; 64%) belong to the order Scolopendromorpha. The second most represented order are the Geophilomorpha with 31% (70 individuals). The Lithobiomorpha are represented by nine specimens (4%) and the Scutigeromorpha are represented by only two specimens (1%).

**Figure 5.**
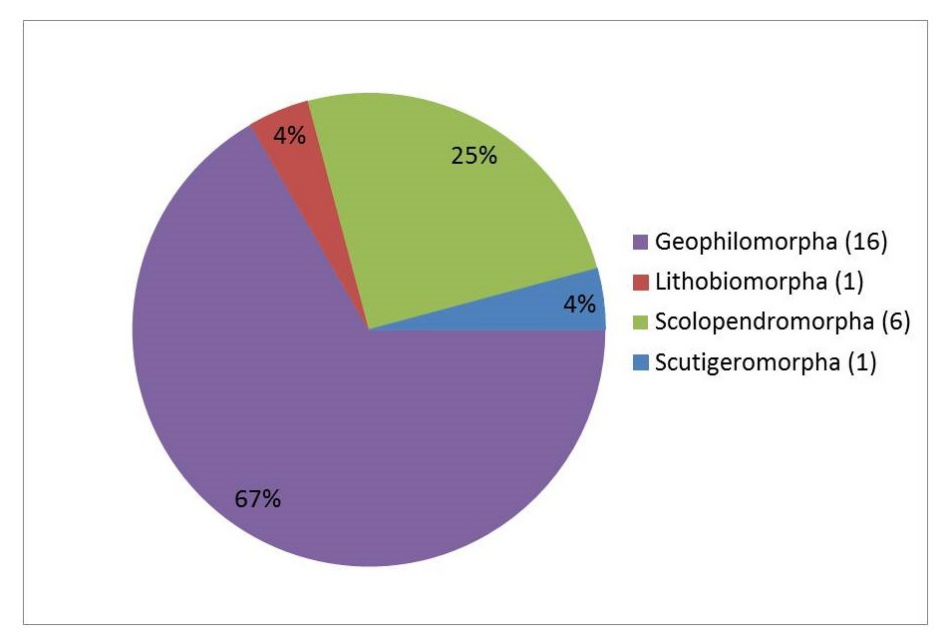
Percentage distribution of the Chilopoda species into the four orders.

About 24 morphospecies (or alternatively species-groups in the case of *Otostigmus*) of Chilopoda were collected in the Halimun-Salak National Park (Fig. 5), with one species each of Scutigeromorpha and Lithobiomorpha. The centipede species diversity was, on the other hand, the highest in the Geophilomorpha (67%; 16 species), followed by the Scolopendromorpha (25%; 6 species). The most common centipede and myriapod species in general was *Otostigmus aff. rugulosus*, with 76 specimens representing 33% of all Chilopoda specimens and 12.3% of all the Myriapoda samples.

### Diplopoda - millipedes

A total of 18 different families and nine orders (Polyxenida, Glomeridesmida, Glomerida, Sphaerotheriida, Siphonophorida, Chordeumatida, Polydesmida, Spirobolida and Spirostreptida) were present in the sample. The most dominant millipede group was the Polydesmida (Table 1). Nearly two-thirds of all Diplopoda (69%; 374 specimens) belonged to the Polydesmida. The superorder Juliformia was represented by the orders Spirobolida (8%; 44 individuals) and Spirostreptida (7%; 37 individuals). Of the Nematophora, only the order Chordeumatida (1%; 4 specimens) was found in the Halimun-Salak National Park, whereas more than 85% of all collected Diplopoda belonged to the Eugnatha. Of the four orders of the Colobognatha, only one order, Siphonophorida (6%; 31 specimens), was found. The infraclass Pentazonia was represented by the orders Glomeridesmida (4%; 25 samples), Glomerida (1%; 6 samples) and Sphaerotheriida (3%; 18 samples). With only four individuals (1%), the Polyxenida was the least represented group in the Halimun-Salak National Park.

**Figure 6.**
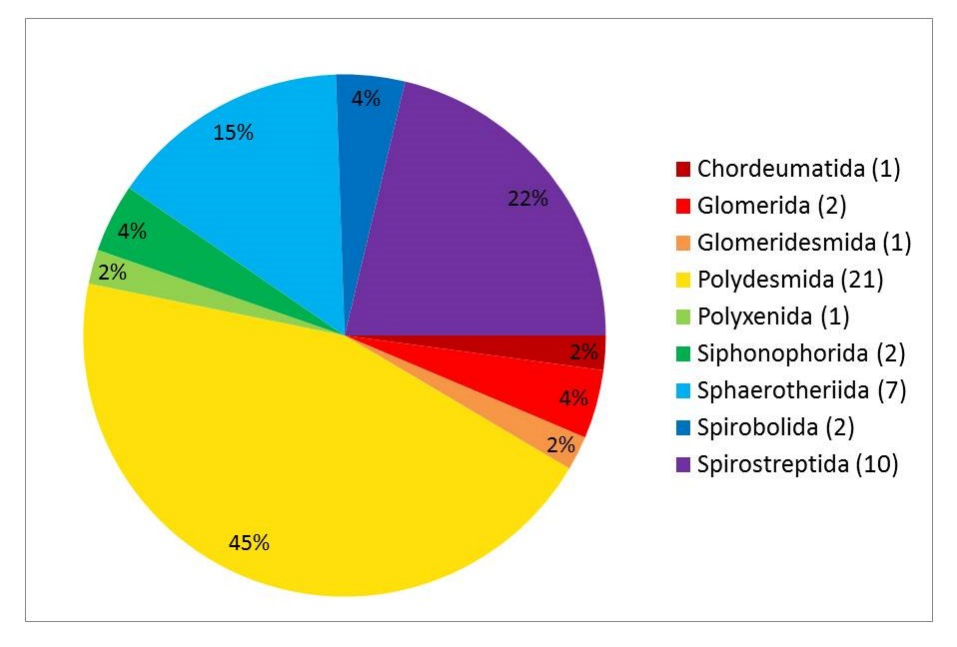
Percentage distribution of the Diplopoda at species-level.

The dominance of the Polydesmida is also reflected in the morphospecies analysis, albeit to a lesser degree (Fig. 6), with 21 (45%) of all observed species. With 10 species (22%), the Spirostreptida showed the second highest species diversity. The Sphaerotheriida also showed high species diversity with seven morphospecies (15%). Glomerida, Siphonophorida and Spirobolida were represented by two species each (4%). The orders Polyxenida, Glomeridesmida and Chordeumatida were present with one morphospecies (2%) each in the Halimun-Salak National Park. The high diversity of the Polydesmida is further reflected at the family- and genus level. Six families, *viz.* Cryptodesmidae, Haplodesmidae, Trichopolydesmidae, Polydesmidae, Platyrhacidae and Paradoxosomatidae, were present, with the vast majority of genera (8 of 14) and morphospecies (13 of 21) belonging to the latter.

### Species accumulation

**Figure 7.**
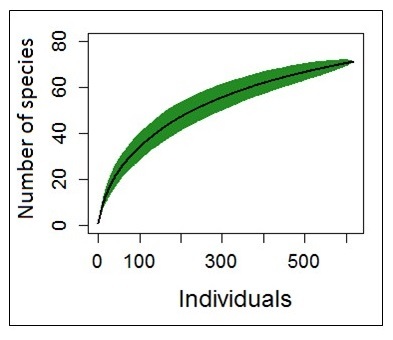
Species accumulation curve for the Halimun-Salak National Park.

**Figure 8.**
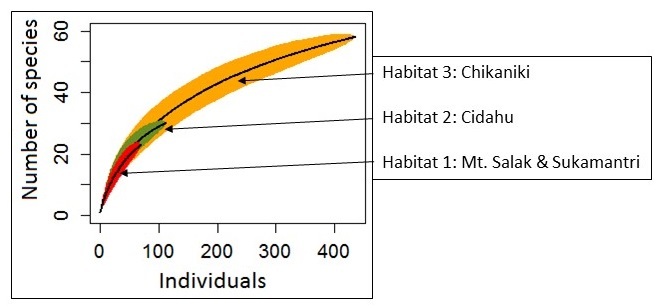
Species accumulation curve for the three different searching locations.

The first curve (Fig. 7) represents all collected samples and species in the entire Halimun-Salak National Park, to determine if the results are meaningful enough to be compared to other Myriapoda faunal compositions. The three other curves (Fig. 8) represent the species accumulation for each habitat. The graph of the forest remnants and plantation (habitat 1) shows no significant indication of saturation (Fig. 8). The one for the former deforested and now regenerating area (habitat 2) seems to become slowly steady although it does not reach the saturation line. Only the amount of collected specimens from the primary rainforest (habitat 3) was enough to determine a saturation line in the species accumulation analysis. The species accumulation curve for all collected samples (Fig. 7) in the Halimun-Salak National Park shows a typical saturation curve with a quick increase of found species per specimen number, with the slope of the curve decreasing as samples were collected until reaching a certain saturation point.

## Discussion

This study represents the first attempt to create a quantitative species list of the centipedes and millipedes of rainforests in tropical Asia during the dry season (in Indonesia). The Halimun-Salak National Park has a total area of 400 km² and houses a highly diverse fauna of vertebrates and invertebrates (Whitten et al. 1997). The national park contains one of the last remaining natural forest habitats on the island of Java (Priyadi et al. 2010). To our knowledge, there are no similar analyses for comparison.

Because no barcoding of the identified samples was performed, it was difficult or even impossible to compare found specimens with already described ones. Therefore, barcoding of the collected samples is mandatory for a proper identification and comparison with other recent taxonomic studies on Myriapoda on Java (e.g Golovatch 2018) or other South-East Asian islands (e.g Zoysa et al. 2016). However, our main goal was to provide an overview of the myriapod faunal composition for a tropical area and not to publish a complete checklist.

Studies from other Asian sites like rural Singapore (Decker 2013) or the Cat Tien National Park in Vietnam (Golovatch et al. 2011) show similar results on the species per order level. In both studies, the Polydesmida were the most diverse group for the millipedes and Polyxenida, Chordeumatida and Glomeridesmida were either absent or the least diverse. With barcode analyses it would be possible to compare the found species with each other to determine if they are the same, or how closely related they are.

Due to the lack of identification keys, all specimens were determined to morphospecies, which might underestimate the actual species diversity in the park. On the one hand, our results yielded the Polydesmida as the most represented and diverse order of the Diplopoda and all Myriapoda, and *Otostigmus aff. rugulosus* as the most common centipede and Myriapoda species in the Halimun-Salak National Park. On the other hand, the underestimation of true morphospecies numbers in this diverse sample will automatically skew the picture towards a lower species diversity, especially in the order Scolopendromorpha.

Although our sampling was mostly qualitative (by hand), smaller species of millipedes such as Polyxenida, Glomeridesmida and Glomerida, as well as many juvenile Polydesmida, were additionally collected. In theory, surface-active millipedes and centipedes have a higher chance to be detected by this collecting method. However, probably due to the prevalent dry season, only a few millipedes were noticed to actively walk around. These were mainly Polydesmida, family Paradoxosomatidae and the spirostreptid genus *Thyropygus*. Most of the collected samples came from digging in the leaf litter and in dead wood, as, for example, the high number of collected Geophilomorpha species attests. The use of sieves and extractors might have increased the chances of finding different species and would definitely affect the percentage distribution of the analysed taxa/specimens.

One of the objectives of this research was to compare the faunal composition between the three selected habitats and assess the impact of human activities on the myriapod fauna in the national park. To be able to perform this comparison in a statistically meaningful way, the amount of collected samples and the number of identified species must correlate. A species accumulation curve gives information if those two data sources correlate and can be compared to other, also correlating data. In our case, only the data from habitat 3, Cikaniki, was significant enough to allow for that. This is most likely due to the simple fact that the collecting effort in Cikaniki was higher than in the other two areas. This made comparison between all three habitats impossible. This could be resolved by future collecting in habitats 1 and 2 during the same season and using the same methodology.

On the other hand, the species accumulation curve of all collected samples correlates with all identified morphospecies in the Halimun-Salak National Park. This proves that our data is significant enough to allow the comparison of our results with other qualitative studies. All samples were collected in the Halimun-Salak National Park and on Java, thus a comparison of the faunal composition of the other islands of Indonesia would also be highly interesting. The newly-gained information about the species diversity represents a first step towards exploring the Myriapoda fauna in one of the most interesting geographic areas on the planet.

## Supplementary Material

Supplementary material 1 Results of all identified samples Data type: Table with identifications to morphospecies
Brief description: Results of all identified Symphyla (family level only), Chilopoda and Diplopoda (to morphospecies level)
File: oo_268331.docx Michael Hilgert

## Figures and Tables

**Figure 1.**
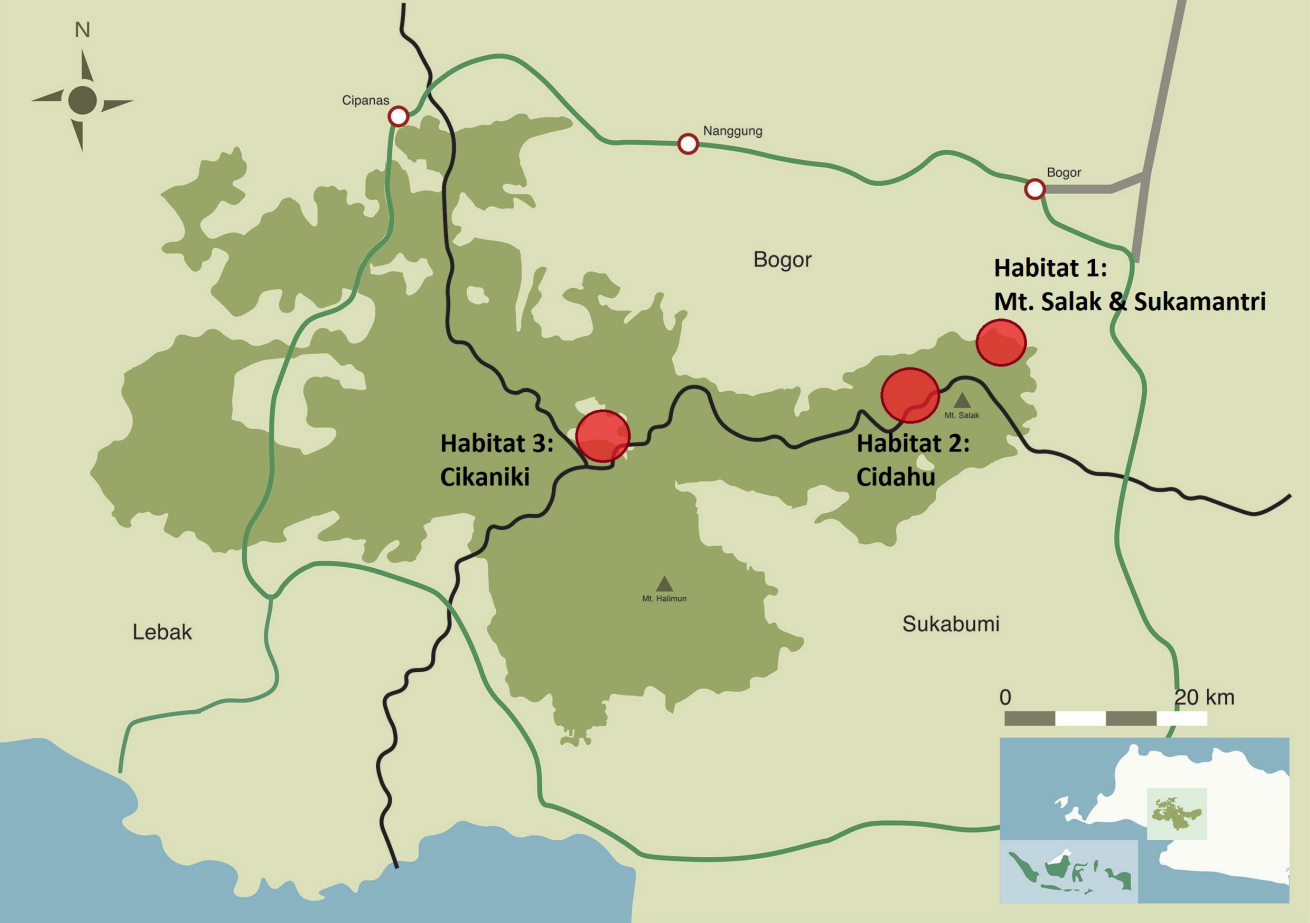
Map of the Halimun-Salak National Park and location of the three collecting sites Mt. Salak and Sukamantri, Cidahu and Cikaniki, modified after Lund and Rachman (2018).

**Figure 2a.**
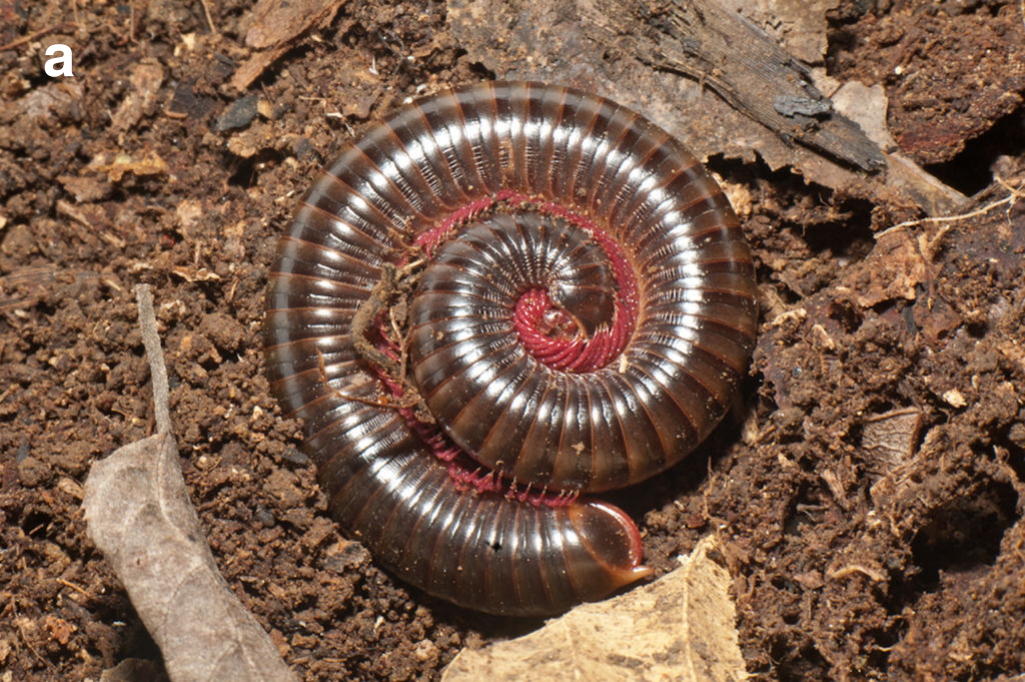
Spirostreptidae, Harpagophoridae, *Thyrophygus* sp.

**Figure 2b.**
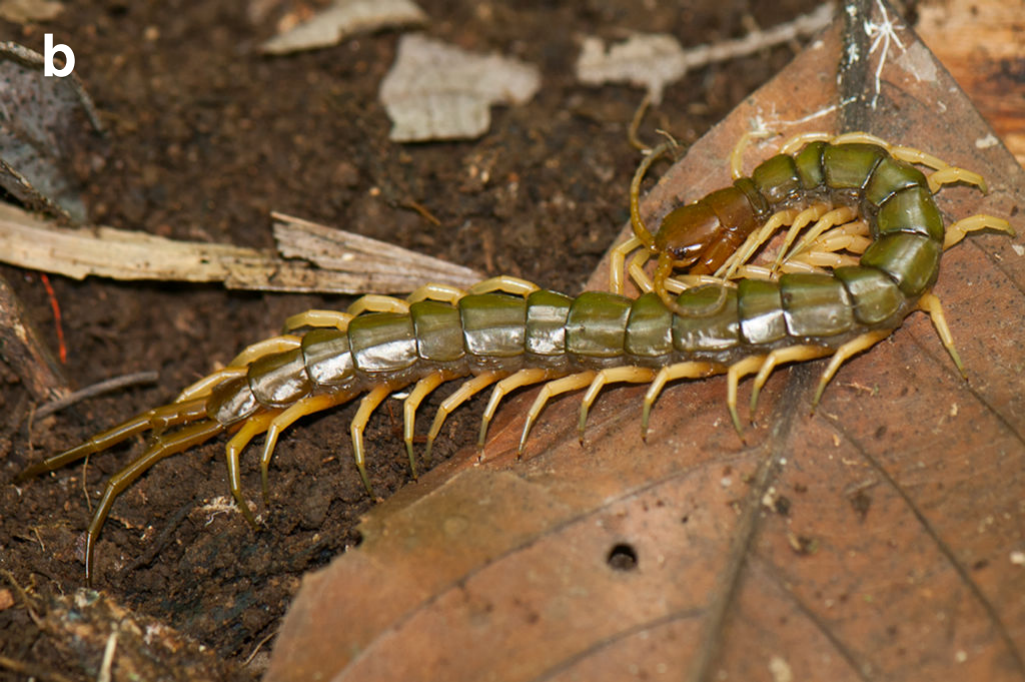
Scolopendromorpha, *Scolopendra subspinipes* Leach, 1815

**Figure 3a.**
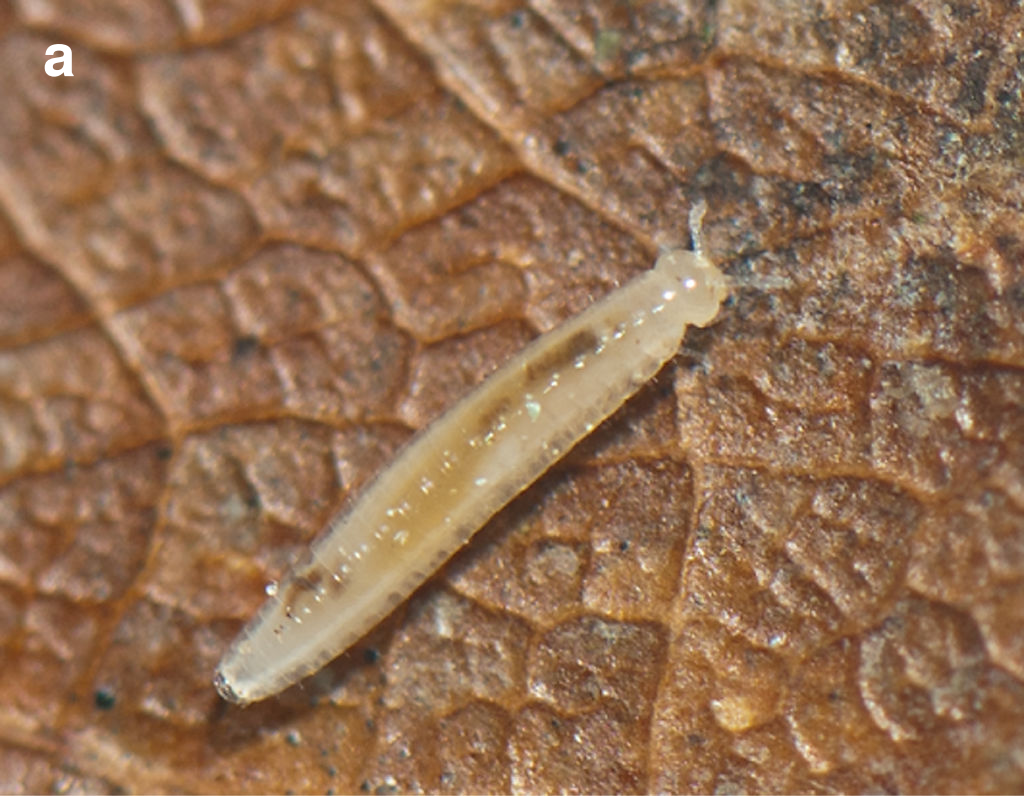
Glomeridesmida, *Glomeridesmus* sp., female

**Figure 3b.**
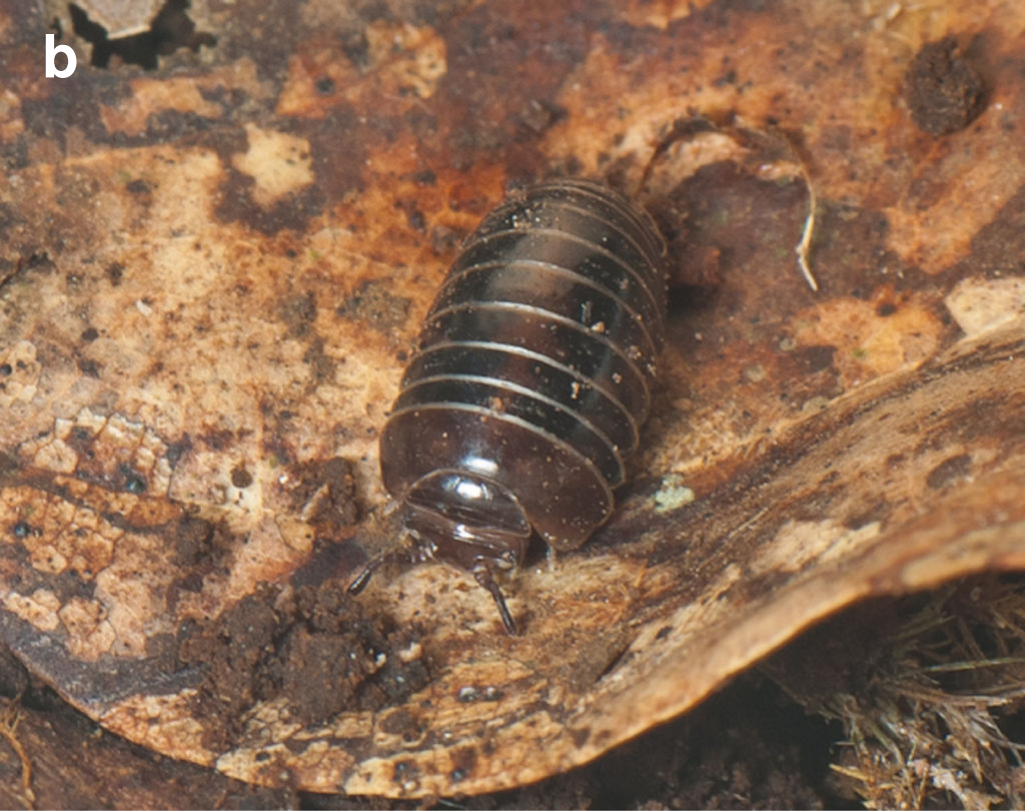
Glomerida, *Hyleoglomeris* sp.

**Figure 3c.**
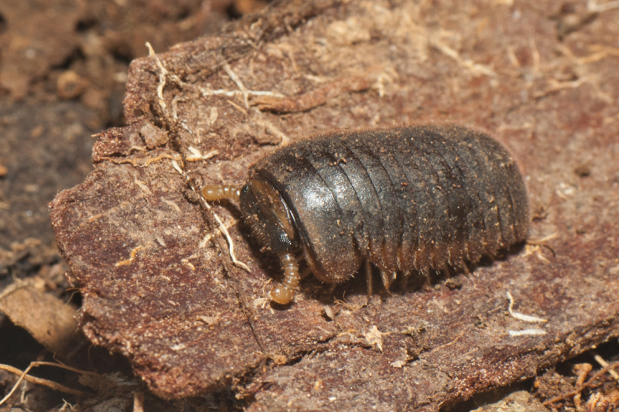
Sphaerotheriida, *Castanotherium* sp.

**Figure 3d.**
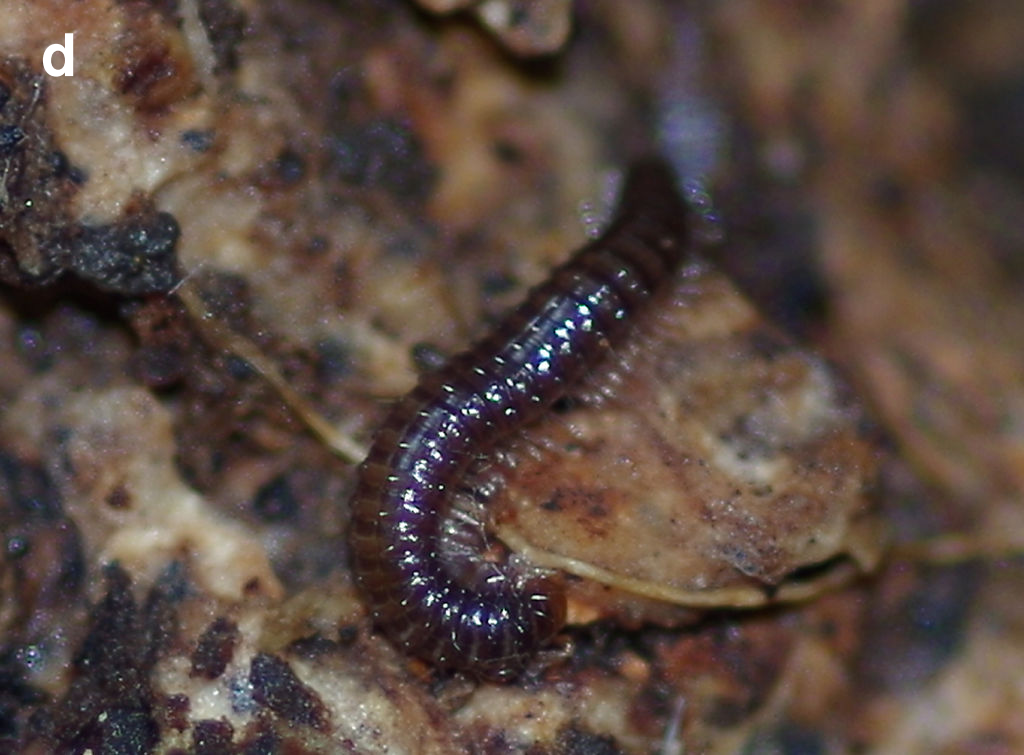
Chordeumatida, family and genus indet.

**Figure 4a.**
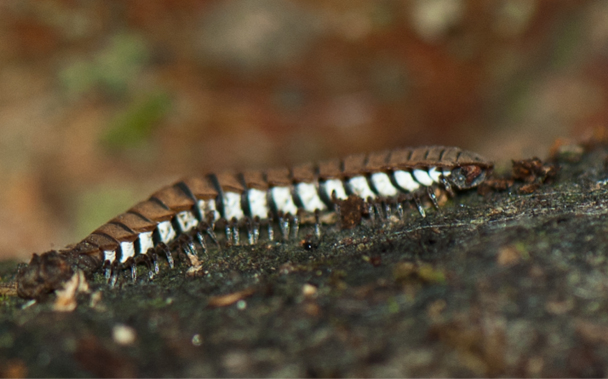
Cryptodesmidae

**Figure 4b.**
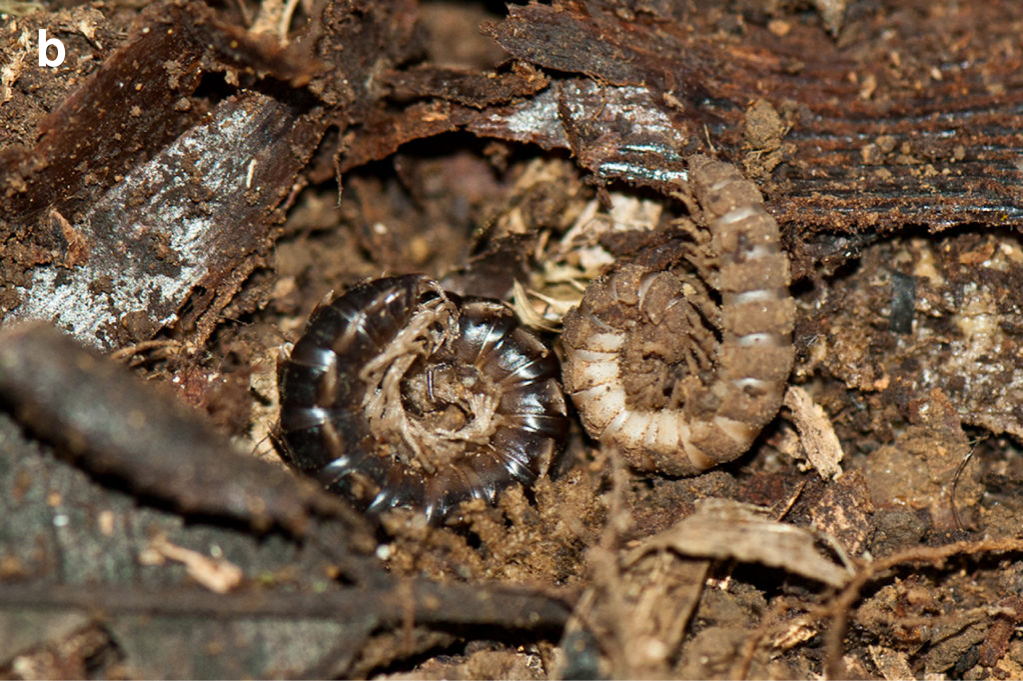
Platyrhacidae, two different species

**Figure 4c.**
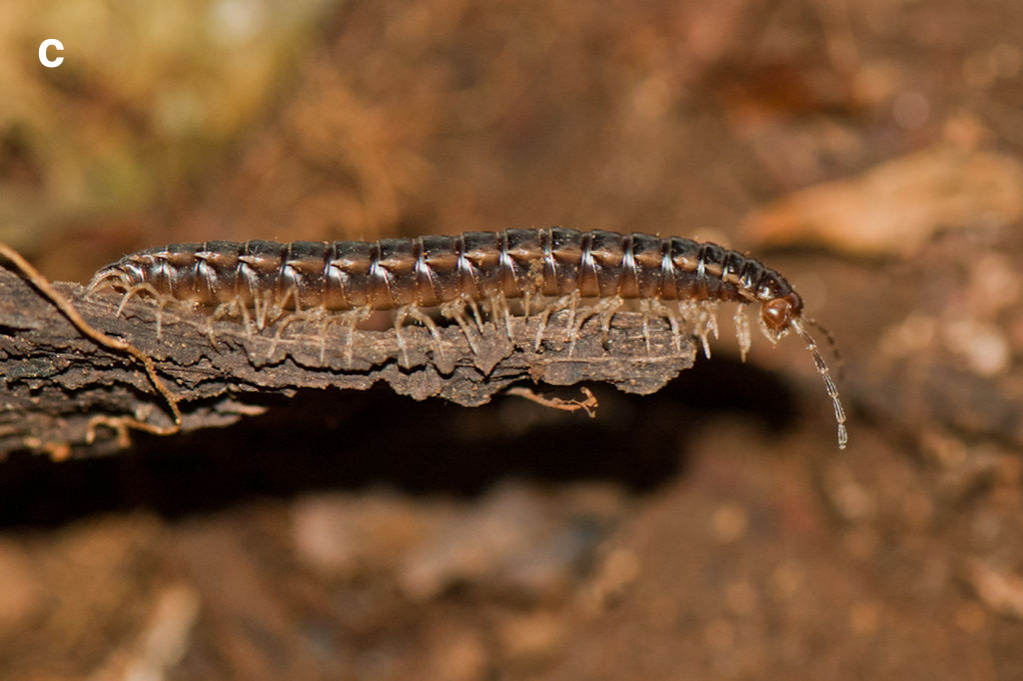
Paradoxosomatidae

**Figure 4d.**
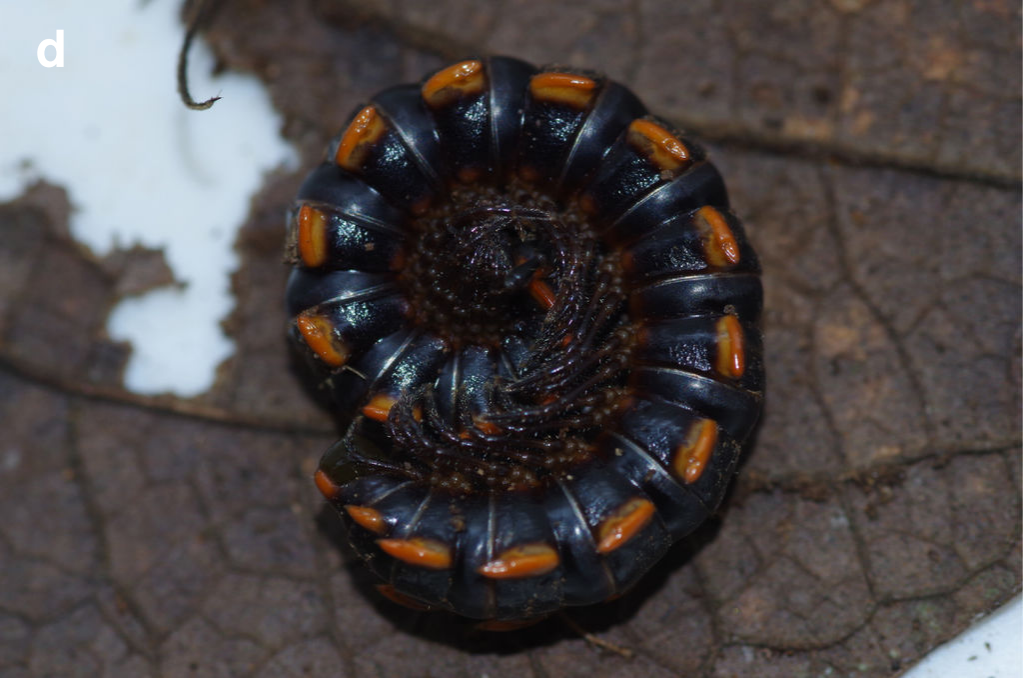
Paradoxosomatidae

**Table 1.** Distribution of all Diplopoda specimens at order-level.

**Order**	**Number of specimens**
Chordeumatida	4
Glomerida	6
Glomeridesmida	25
Polydesmida	374
Polyxenida	4
Siphonophorida	31
Sphaerotheriida	18
Spirobolida	44
Spirostreptida	37
**Total**	543
