# Supplementary material for: The Myriapoda of Halimun-Salak National Park (Java, Indonesia): overview and faunal composition
Source: Biodivers Data J. 2019 Apr 15;7:e32218. doi: 10.3897/BDJ.7.e32218 (PMC6477843; doi:10.3897/BDJ.7.e32218)
Supplement: Supplementary material 1 — Results of all identified samples [file bdj-07-e32218-s001.docx]

**Class: Symphyla**

| **Order** | **Family** | **Sub-family** | **Genus** | **Species/ Species-group/ Morphospecies** | **Count** |
| --- | --- | --- | --- | --- | --- |
|  | Scutigerellidae |  |  |  | 27 |

**Class: Chilopoda**

| **Order** | **Family** | **Sub-family** | **Genus** | **Species/ Species-group/ Morphospecies** | **Count** |
| --- | --- | --- | --- | --- | --- |
| Scutigeromorpha | Scutigeromorpha Family 1 |  | Scutigeromorpha Family 1 Genus A | Scutigeromorpha Family 1 Genus A spec. 1 | 2 |
| Lithobiomorpha | Lithobiomorpha Family 1 |  | Lithobiomorpha Family 1 Genus A | Lithobiomorpha Family 1 Genus A spec. 1 | 9 |
| Scolopendromorpha | Cryptopidae | Cryptopinae | Cryptops | Cryptops spec. 1 | 45 |
|  | Scolopocryptopidae | Scolopocryptopinae | Scolopocryptops | Scolopocryptops spec. 1 | 16 |
|  | Scolopendridae | Scolopendrinae | Scolopendra | S. subspinipes | 1 |
|  |  | Otostigminae | Ethmostigmus | Ethmostigmus spec. 1 | 4 |
|  |  |  | Otostigmus | Oto. orientalis group | 1 |
|  |  |  |  | Oto. rugulosus group | 76 |
| Geophilomorpha | Ballophilidae |  | Ballophilidae Genus A | Ballophilidae Genus A spec. 1 | 1 |
|  | Mecistocephalidae |  | Mecistocephalidae Genus A | Mecistocephalidae Genus A spec. 1 | 2 |
|  |  |  |  | Mecistocephalidae Genus A spec. 2 | 3 |
|  |  |  |  | Mecistocephalidae Genus A spec. 3 | 1 |
|  |  |  |  | Mecistocephalidae Genus A spec. 4 | 3 |
|  |  |  |  | Mecistocephalidae Genus A spec. 5 | 1 |
|  |  |  |  | Mecistocephalidae Genus A spec. 6 | 3 |
|  |  |  | Mecistocephalidae Genus B | Mecistocephalidae Genus B spec. 1 | 1 |
|  |  |  |  | Mecistocephalidae Genus B spec. 2 | 1 |
|  |  |  |  | Mecistocephalidae Genus B spec. 3 | 2 |
|  |  |  |  | Mecistocephalidae Genus B spec. 4 | 7 |
|  |  |  |  | Mecistocephalidae Genus B spec. 5 | 6 |
|  |  |  |  | Mecistocephalidae Genus B spec. 6 | 3 |
|  |  |  |  | Mecistocephalidae Genus B spec. 7 | 1 |
|  |  |  |  | Mecistocephalidae Genus B spec. 8 | 1 |
|  |  |  |  | Mecistocephalidae Genus B spec. 9 | 1 |

**Class: Diplopoda**

| **Order** | **Sub-order** | **Family** | **Genus** | **Species/ Species-group/ Morphospecies** | **Count** |
| --- | --- | --- | --- | --- | --- |
| Polyxenida |  | Polyxenidae | Polyxenidae Genus A | Polyxenidae Genus A spec. 1 | 4 |
| Glomeridesmida |  | Glomeridesmidae | Glomeridesmus | Glomeridesmus spec. 1 | 25 |
| Glomerida |  | Glomeridae | Hyleoglomeris | H. spec. 1 | 4 |
|  |  |  |  | H. spec. 2 | 2 |
| Sphaerotheriida |  | Zephroniidae | Castanotherium | C. spec. 1 | 1 |
|  |  |  |  | C. spec. 2 | 5 |
|  |  |  |  | C. spec. 3 | 3 |
|  |  |  |  | C. spec. 4 | 1 |
|  |  |  |  | C. spec. 5 | 2 |
|  |  |  |  | C. spec. 6 | 3 |
|  |  |  |  | C. spec. 7 | 3 |
| Siphonophorida |  | Siphonophoridae | Siphonophoridae Genus A | Siphonophoridae Genus A spec. 1 | 26 |
|  |  | Siphonorhinidae | Siphonorhinus | Siphonorhinus spec. 1 | 5 |
| Chordeumatida |  | Chordeumatida Family 1 | Chordeumatida Family 1 Genus A | Chordeumatida Family 1 Genus A spec. 1 | 4 |
| Polydesmida |  | Cryptodesmidae | Ophrydesmus | Ophry. weberi | 45 |
|  |  |  |  | Ophry. spec. 1 | 20 |
|  |  | Haplodesmidae | Helodeus | Helodeus spec. 1 | 6 |
|  |  |  | Haplodesmidae Genus A | Haplodesmidae Genus A spec. 1 | 1 |
|  |  | Paradoxosomatidae | Paradoxosomatidae Genus A | Paradoxosomatidae Genus A spec. 1 | 16 |
|  |  |  |  | Paradoxosomatidae Genus A spec. 2 | 1 |
|  |  |  |  | Paradoxosomatidae Genus A spec. 3 | 1 |
|  |  |  |  | Paradoxosomatidae Genus A spec. 4 | 1 |
|  |  |  | Paradoxosomatidae Genus B | Paradoxosomatidae Genus B spec. 1 | 7 |
|  |  |  |  | Paradoxosomatidae Genus B spec. 2 | 4 |
|  |  |  | Paradoxosomatidae Genus C | Paradoxosomatidae Genus C spec. 1 | 40 |
|  |  |  | Paradoxosomatidae Genus D | Paradoxosomatidae Genus D spec. 1 | 7 |
|  |  |  | Paradoxosomatidae Genus E | Paradoxosomatidae Genus E spec. 1 | 3 |
|  |  |  |  | Paradoxosomatidae Genus E spec. 2 | 1 |
|  |  |  | Paradoxosomatidae Genus F | Paradoxosomatidae Genus F spec. 1 | 26 |
|  |  |  | Paradoxosomatidae Genus G | Paradoxosomatidae Genus G spec. 1 | 1 |
|  |  |  | Paradoxosomatidae Genus H | Paradoxosomatidae Genus H spec. 1 | 2 |
|  |  | Platyrhacidae | Platyrhacidae Genus A | Platyrhacidae Genus A spec. 1 | 62 |
|  |  |  |  | Platyrhacidae Genus A spec. 2 | 2 |
|  |  | Polydesmidae | Polydesmidae Genus A | Polydesmidae Genus A spec. 1 | 10 |
|  |  | Trichopolydesmidae | Trichopolydesmidae Genus A | Trichopolydesmidae Genus A spec. 1 | 6 |
| Spirobolida |  | Pachybolidae | Trigoniulus | Tri. spec. 1 | 35 |
|  |  |  |  | Tri. spec. 2 | 9 |
| Spirostreptida |  | Spirostreptida Family 1 | Spirostreptida Family 1 Genus A | Spirostreptida Family 1 Genus A spec. 1 | 1 |
|  | Cambalidea | Cambalopsidae | Glyphiulus | Glyphiulus spec. 1 | 3 |
|  |  | Cambalidea Family 1 | Cambalidea Family 1 Genus A | Cambalidea Family 1 Genus A spec. 1 | 3 |
|  |  | Harpagophoridae | Thyropygus | Thy. spec. 2 | 5 |
|  |  |  |  | Thy. spec. 4 | 1 |
|  |  |  |  | Thy. spec. 5 | 7 |
|  |  |  |  | Thy. spec. 6 | 8 |
|  |  |  |  | Thy. spec. 7 | 2 |
|  |  |  |  | Thy. spec. 8 | 1 |
|  |  |  |  | Thy. spec. 9 | 1 |
